# 1627. Antibiotic Use and Clinical Outcomes in Peripartum Infections

**DOI:** 10.1093/ofid/ofad500.1462

**Published:** 2023-11-27

**Authors:** Emily A Siegrist, Joseph Sassine

**Affiliations:** OU Health, Oklahoma City, Oklahoma; University of Oklahoma Health Sciences Center, Oklahoma City, Oklahoma

## Abstract

**Background:**

Peripartum infections are a leading cause of maternal morbidity and mortality. Historically, these infections have been treated with combinations of ampicillin and gentamicin with or without clindamycin. However, resistance in anaerobic and gram-negative bacteria to these antimicrobials has been increasing nationally. There is limited modern data evaluating the incidence and outcomes of chorioamnionitis and endometritis, especially in the context of modern resistance patterns and antibiotic regimens.

**Methods:**

This is a single-center, retrospective study at an academic medical center. The primary objective is to describe antibiotic use and outcomes in patients diagnosed with chorioamnionitis or endometritis. Patients were identified by ICD codes using Vigilanz software. Patients with a coded diagnosis for chorioamnionitis or endometritis in 2022 were included and stratified according to highest level of care. Chi-square and Fisher’s exact test were used for categorical variables, Mann-Whitney test for continuous variables.

**Table 1:**
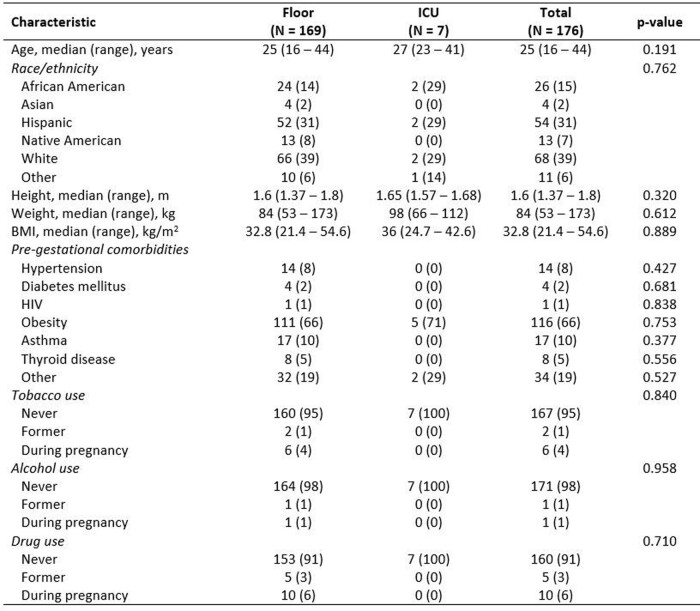
Baseline Demographics

**Table 2:**
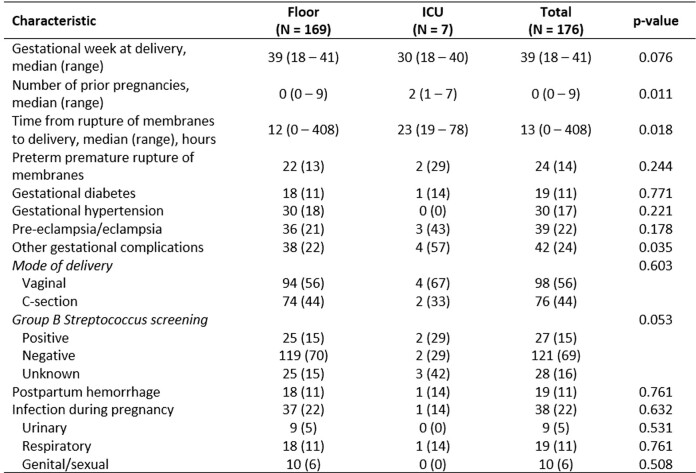
Pregnancy and Delivery Characteristics

**Results:**

We included 176 patients, indicating 4.6% of deliveries were complicated by a peripartum infection. Seven patients (4%) required ICU level of care. These patients were more likely to be multiparous, have longer time from rupture of membranes to delivery, have vaginal discharge, and had more pronounced leukocytosis with left shift. The most common organism isolated was *Escherichia coli* and only piperacillin-tazobactam (TZP) was active against all isolates. Patients admitted to the ICU were more likely to have received metronidazole, TZP, vancomycin or a cephalosporin, to have switched antibiotics and to have longer days of therapy. Only one patient from the ICU group passed away for causes unrelated to the infection.

**Table 3:**
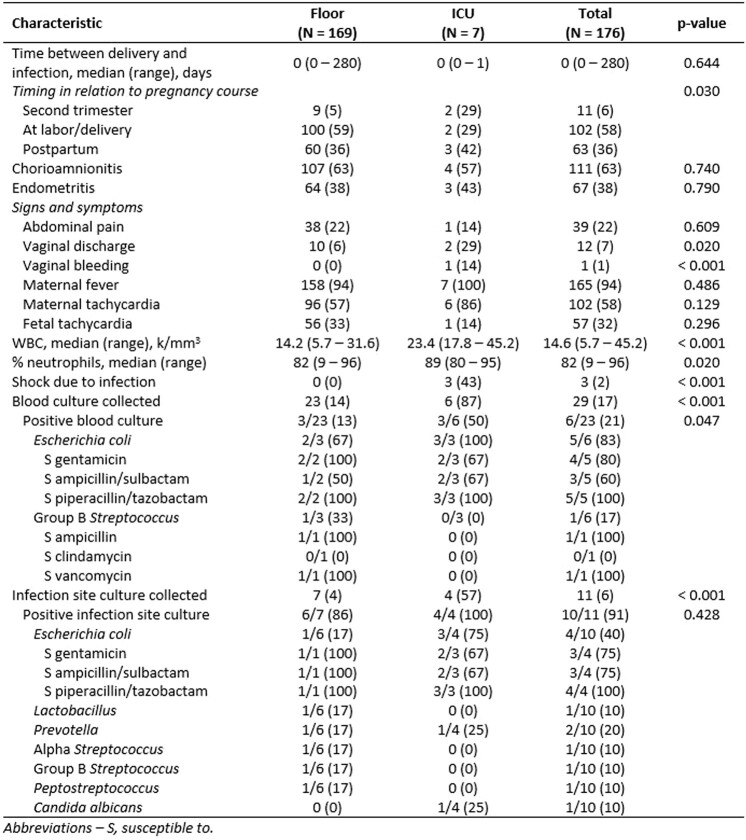
Peripartum Infection Characteristics

**Table 4:**
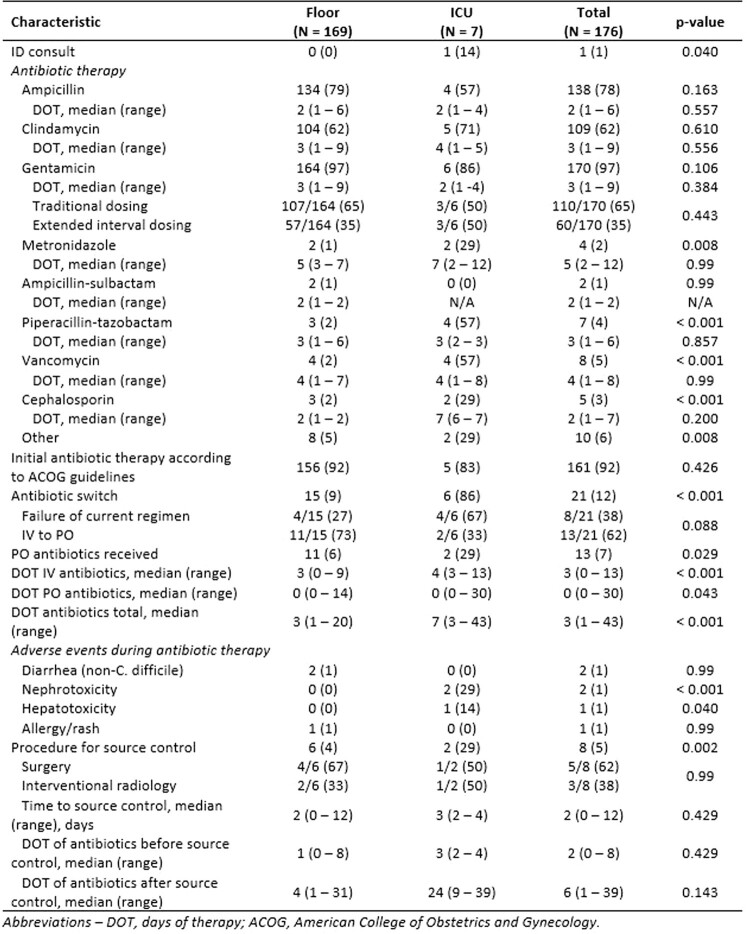
Peripartum Infection Management

**Table 5:**
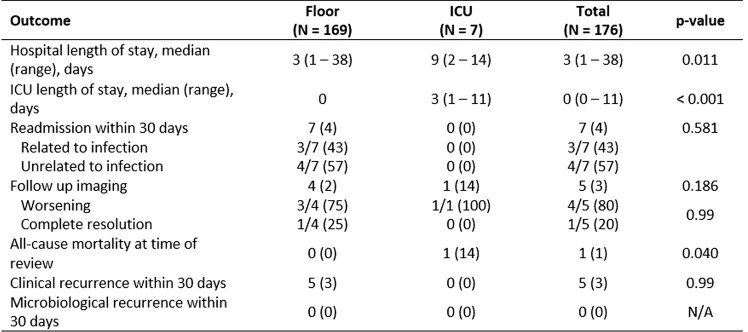
Outcomes

**Conclusion:**

Peripartum infections are common, and most patients improve on short courses of clindamycin, gentamicin, and ampicillin. Historical antibiotic regimens may not have desired activity against *E. coli* isolates, and clinicians may consider use of TZP for patients failing to improve on standard therapy. Larger studies are needed to establish a more specific diagnosis, validate risk factors for severity, and explore the efficacy of updated antimicrobial regimens.

**Disclosures:**

**All Authors**: No reported disclosures

